# Automated Measurements of Tooth Size and Arch Widths on Cone-Beam Computerized Tomography and Scan Images of Plaster Dental Models

**DOI:** 10.3390/bioengineering12010022

**Published:** 2024-12-29

**Authors:** Thong Phi Nguyen, Jang-Hoon Ahn, Hyun-Kyo Lim, Ami Kim, Jonghun Yoon

**Affiliations:** 1 Department of Mechanical Design Engineering, Hanyang University, 222, Wangsimni-ro, Seongdongsu, Seoul 04763, Republic of Korea; npthong2511@hanyang.ac.kr (T.P.N.);; 2 BK21 FOUR ERICA-ACE Center, Hanyang University, Ansan 15588, Gyeonggi-do, Republic of Korea; 3 Department of Orthodontics, Gwangmyeong Hospital, Chungang University, 110, Deokan-ro, Gwangmyeong 07440, Gyeonggi-do, Republic of Korea; ajh0225@cauhs.or.kr; 4 Department of Mechanical Engineering, Hanyang University, 55, Hanyangdaehak-ro, Sangnok-gu, Ansan 15588, Gyeonggi-do, Republic of Korea; 5 Seoul Ami Orthodontic Private Practice, 22, Harmony-ro, 178 Beon-gil, Yeonsu-gu, Incheon 22011, Republic of Korea

**Keywords:** cone-beam computerized tomography, Mask-RCNN, teeth segmentation, teeth identification, key points detection

## Abstract

Measurements of tooth size for estimating inter-arch tooth size discrepancies and inter-tooth distances, essential for orthodontic diagnosis and treatment, are primarily done using traditional methods involving plaster models and calipers. These methods are time-consuming and labor-intensive, requiring multiple steps. With advances in cone-beam computerized tomography (CBCT) and intraoral scanning technology, these processes can now be automated through computer analyses. This study proposes a multi-step computational method for measuring mesiodistal tooth widths and inter-tooth distances, applicable to both CBCT and scan images of plaster models. The first step involves 3D segmentation of the upper and lower teeth using CBCT, combining results from sagittal and panoramic views. For intraoral scans, teeth are segmented from the gums. The second step identifies the teeth based on an adaptively estimated jaw midline using maximum intensity projection. The third step uses a decentralized convolutional neural network to calculate key points representing the parameters. The proposed method was validated against manual measurements by orthodontists using plaster models, achieving an intraclass correlation coefficient of 0.967 and a mean absolute error of less than 1 mm for all tooth types. An analysis of variance test confirmed the statistical consistency between the method’s measurements and those of human experts.

## 1. Introduction

Orthodontic treatment aims to rearrange the patient’s teeth in proper inter-digitation with correct overjet and overbite [1], which is remarkably complicated owing to biological limitations. There are limited options to achieve ideal results without loss of teeth by extractions or gain by adding composites. Therefore, orthodontic diagnosis and treatment planning are important processes for orthodontists to provide the most effective and efficient predictions for their patients [2]. These processes include determining the anterior–posterior relationships of the skeleton, asymmetry of the face, and degree of incisor inclination based on multiple data types such as lateral cephalograms, posterior–anterior cephalograms, and three-dimensional (3D) cone beam computed tomography (CBCT) images [3]. Moreover, the Bolton ratio, which represents the tooth size proportion of maxillary teeth to mandibular teeth and is measured on dental models, is the most popular feature representing the status of tooth alignment correction [4,5]. If there is a significant difference in the Bolton ratio of maxillary and mandibular teeth, it will be challenging to correct teeth inter-digitation between the upper and lower arch. Maxillary and mandibular teeth must be proportional in size to achieve proper occlusion with normal overjet and overbite [6]. With the development of artificial intelligence (AI) and the trend of applying it to various fields, there have been applications of AI in the field of orthodontics, such as dental diagnostics [7,8,9], cephalometric analysis [10,11,12], determination of skeletal age [13,14,15], orthognathic surgery decision making and planning [16,17,18], and patient monitoring [19,20,21].

Traditionally, orthodontists measure teeth size using plaster models with a caliper. In particular, the plaster model is manufactured by taking the impression of a patient’s mouth using an impression material and then pouring dental plaster into the negative space, which requires a significant amount of time and effort [22]. This disadvantage remains in the measurement process, where the size of every tooth is manually and sequentially measured on the plaster models using a Vernier caliper [23]. Not only are there clinically significant errors in the method reported by Hunter et al. [24] but also the inconvenience of these processes leads to wasting time and human effort. Therefore, there is a need to develop alternative approaches.

Subsequently, photocopy, hologram, and video capture images have been introduced as solutions to reduce the errors [25,26,27,28].

With the development of digital techniques, three-dimensional (3D) data types can capture the geometric characteristics of the teeth for the diagnosis process. An intraoral scanner, which can capture the 3D surface of the hard and soft tissues in an oral cavity, is a good choice to replace the traditional method of using plaster models. The output data, in the form of a surface or point cloud, can be manipulated via the software that allows users to observe, annotate by clicking, and measure the distance between selected points using software [29]. The benefits of digitalization compared to traditional methods include increased efficiency owing to easy usage, immunity to physical damage, and reduced storage space requirements [30,31]. In spite of these advantages, orthodontists still require time to manipulate a program to define the key-points to measure tooth size, which requires skills and knowledge.

The CBCT technique, which was introduced to the dental profession between 2001 and 2004 by the NewTom 9000 (QR, Verona, Italy), CB MercuRay (Hitachi Medical Systems America, Twinsburg, OH, USA), and I-CAT (Imaging Sciences, Hatfield, PA, USA), allows users to observe not only the 3D geometry of human skulls but also the inner structures and tissues. However, there is inevitable noise caused by the soft tissue such as flesh and skin, which limits the accuracy in extracting the teeth as parts of interest from the raw data.

Artificial intelligence (AI) techniques have been applied in dentistry and orthodontics to detect landmarks on two dimensional (2D) lateral cephalograms [32,33,34,35]. With the developments in 3D data types, there have been studies on the detection of landmarks on CBCT images. Zheng et al. [36] presented a local radial basis function collocation method (LRBFCM) to analyze a 3D tooth domain built using CBCT data. Wang et al. [37] provided a method for extracting lateral cephalograms from CBCT data and detecting landmarks on it. Ahn et al. [38] introduced a fully automated method to measure 13 parameters according to the key points located on CBCT by applying a region-based convolutional neural network (R-CNN), namely Mask R-CNN, and a decentralized convolutional neural network (CNN). Jang et al. [39] proposed a method for individual 3D tooth identification and segmentation by combining the segmentation results from axial and panoramic views.

Although several studies have applied deep learning to analyze 3D digital data, the need to automate the measurement process of inter-arch tooth size discrepancy has not been accomplished. This is because of the high complexity that this function requires, which not only successfully segments and separates the upper and lower teeth from the raw data under noise and overlapping situations but also efficiently detects, identifies, and measures individual tooth size in a 3D manner.

This study aims to provide a fully automated solution for these needs by developing a systematic deep learning-based method to measure multiple distance parameters using both CBCT and scan images of a plaster dental model. Moreover, a systematic method is proposed to segment not only the 3D upper and lower teeth from the raw CBCT but also the teeth section from the scan images of the plaster dental model. The identification of individual teeth and 3D tooth sizes based on Bolton laws [40] are also automatically estimated. To evaluate the clinical applicability of the developed method, a comparison was made between the results of the proposed method and those obtained with manual measurement using plaster dental models conducted by two orthodontists, which are the current gold standard.

## 2. Materials

### 2.1. Parameters for Measurement

**Figure 1:**
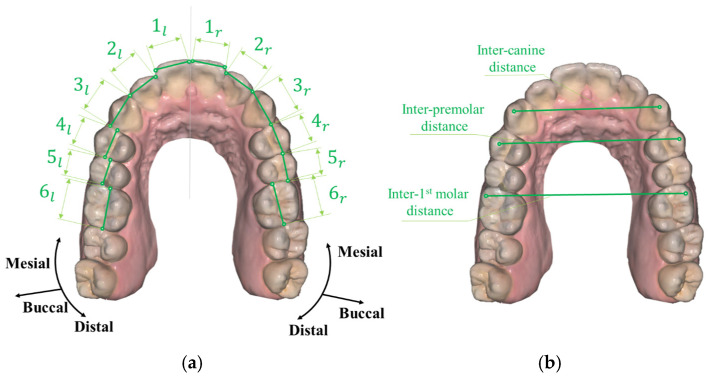
(**a**) Tooth size defined as mesio-distal width from central incisor to 1st molar on left and right sides. (**b**) Arch widths on canine, 1st premolar, and 1st molar.

The first parameter is a mesio-distal tooth width defined as tooth size. To define the tooth size on the axial view, it is necessary to clarify the designated directions according to the tooth location. In this study, the mesio-distal widths of 12 teeth from the right 1st molar to the left 1st molar were required for calculating the Bolton ratio as shown in Figure 1. Tooth size is the width of a tooth along the mesial/distal direction where the mesial and distal directions represent the direction toward and away from the midline of the face respectively. Both CBCT and scan images of plaster dental models were used to measure these parameters.

The second parameter is maxillary arch width [41]. In this study, three maxillary arch widths were measured as shown in Figure 1b, including:

1. Inter-canine distance: the linear distance between canine cusp tips.
2. Inter-premolar distance: the linear distance between the buccal cusp tips of the 1st premolars.
3. Inter–first molar distance: the distance between the mesio-buccal cusp tips of the first molars.

In terms of the 2nd parameter, sufficient resolution of the tooth crown is required to accurately partition these sections and locate the position of the cusp tips for distance measurements. Only scan images of plaster dental models are used to measure this parameter because of the low resolution of CBCT depicting rough tooth crowns.

### 2.2. Dataset

This study was reviewed and approved by the Institutional Review Board of Kangnam Sacred Heart Hospital, Hallym University (2022-04-018). A total of 200 CBCT data points collected at the NHP from 200 orthodontic patients who visited the Department of Orthodontics, Hallym University, Kangnam Sacred Heart Hospital, Seoul, Republic of Korea, were used to build the dataset for this study. Also, 200 plaster models were collected by taking alginate impressions. This collection process included informed consent from the patients. The exclusion criteria included a history of orthodontic treatment, orthognathic surgery, and prosthetic treatment of the upper or lower anterior teeth.

Each CBCT case contained 3D scanning data with 576 slices at a resolution of 768 × 768 × 576 pixels and slice thickness of 0.3 mm. Full field-of-view (23 × 17 cm) images were taken with an I-CAT CBCT scanning machine (KaVo Dental GmbH, Biberach, Germany) using the operational parameters of 120 Kv, 37.1 mA, a voxel size of 0.3 mm, and a scan time of 8.9 s. The capture condition was standardized with the patient’s eyes asked to focus on a 40 × 50 cm mirror, which was hung on the wall 1.5 m from the patient’s position, after exercising their head up and down according to Solow and Tallgren’s method [42] before operating a CBCT scan machine.

A high-resolution laser scanner (3Shape Trios3, Copenhagen, Denmark) was used to obtain 3D digital dental models with sufficient quality in the PLY file format using 200 plaster models.

## 3. Methods

The proposed method consists of the following three steps: First, the 3D teeth images of both the upper and lower jaws were extracted from the raw CBCT data as input data by combining the segmentation results along the sagittal direction and from the panoramic view. The teeth section was segmented from the gum section based on color classification in case of the scan images of plaster dental models as the input data. Second, individual tooth position and identification are sequentially detected and defined based on the segmentation results of the maximum intensity projection (MIP) and face midline estimation results. Third, the key-points are detected to measure tooth size by applying a decentralized CNN [43,44,45,46].

### 3.1. 3D Upper and Lower Teeth Segmentation from CBCT Data

The original range, which is the full-scan of a patient’s skull, is narrowed to the teeth region between the A-point, B-point, and Ar-point, which are detected using the method developed by Ahn et al. [38]. The narrowed CBCT images X with voxel grid $Ω:=\left\{\left(x,y,z\right)\in {\mathbb{N}}^{3}\;:1\leq x\leq N_{x},\;1\leq y\leq N_{y},\;1\leq z\leq N_{z}\right\}$, where $N_{x}$, $N_{y}$, and $N_{z}$ are the voxel sizes in directions x (frontal axis), y (sagittal axis), and z (axial axis), respectively, is considered as the official 1st input type in this study. The value X(x,y,z) represents the Hounsfield units in CBCT images at voxel position (x,y,z).

Step 1-1: Along the sagittal direction, the 3D segmentation process was performed by applying the re-trained Mask R-CNN [47] on every slice. The reason for choosing the sagittal direction for teeth segmentation is to generate the most convenient circumstance for separating the upper and lower teeth. This effect comes from an optimized viewpoint, which yields an uncomplicated definition of the boundary between the upper and lower teeth without requiring a perspicuous empty space between the two jaws which is not regularly available. The loss function for the Mask R-CNN is defined in Equation (1).

(1) $$L=L_{cls}+L_{box}+L_{mask}$$

where L is the total loss, $L_{cls}$ is the loss of classification, $L_{box}$ is the loss of the bounding box regression, and $L_{mask}$ is the loss of the binary mask, which is defined in Equation (2).

(2) $$L_{mask}=-\frac{1}{m^{2}}\underset{1\leq i,\;j\leq m}{\sum }\left[y_{ij}log{\hat{Y}}_{ij}^{k}+\left(1-Y_{ij}\right)\log\left(1-{\hat{Y}}_{ij}^{k}\right)\right]$$

where $Y_{ij}$ is the label of cell (i,j) in the true mask for the region of size m×m, and ${\hat{Y}}_{ij}^{k}$ is the predicted value of the same cell for the ground truth class k.

To distinguish between the upper and lower jaws, the segmented mask $\;{M_{sagittal}}_{n}\left(s\right)$ in slice $s\leq N_{y}$ is represented by the centre point $p_{\;{M_{sagittal}}_{n}\left(s\right)}$ of the detected bounding box $\left(x_{n}\left(s\right),z_{n}\left(s\right),h_{n}\left(s\right),w_{n}\left(s\right)\right)$. The coordinates of the representative point $p_{\;{M_{X_{upper}}}_{n}\left(s\right)}$ are stated in Equation (3).

(3) $$p_{\;{M_{sagittal}}_{n}\left(s\right)}\left(x,y,z\right)=\left(x_{n}\left(s\right)+\frac{w_{n}\left(s\right)}{2},s,z_{n}\left(s\right)+\frac{h_{n}\left(s\right)}{2}\right)$$

Step 1-2: For the axial view, the mean intensity projection along this direction is calculated, which is used for detecting the dental arch curve of jaw $C_{X}$ using the technique proposed in a previous study conducted by Ahn et al. [38]. The obtained curve can be expressed as:

(4) $$C_{X}=\left\{r\left(c\right)=\left(x\left(c\right),y\left(c\right)\right):\;1\leq c\leq N_{c}\right\}$$

where $N_{c}$ is the number of points on the curves and is the width of the panoramic image as well.

A panoramic image is extracted using the following based on the obtained dental curve results:

(5) $$P_{X}\left(c,z\right)=\int_{-w}^{w}X\left(r\left(c\right)+tn\left(c\right),z\right)dt$$

where c is the index of point in the dental arch described in Equation (4), $r\left(c\right)\in C_{X}$, n(c) is the unit normal vector at r(c), and w is the considered range from the dental arch to the normal vector n(c).

Step 1-3: The tooth segmentation is first performed on the panoramic view by applying Mask R-CNN. The masks obtained are split into upper $\;{M_{pano}}_{upper}$ and lower ${M_{pano}}_{lower}$ masks, which represent the upper and lower teeth, respectively; linear regression is applied to estimate the boundary between jaws [38].

Second, representative points $p_{\;{M_{sagittal}}_{n}\left(s\right)}$, which represent segmented masks along the sagittal axis, are projected on the panoramic image as $p_{pano}^{sagi}$, similar to the conversion process for the panoramic view stated in Equation (5).

(6) $$p_{pano}^{sagi}=\left\{p\left(r\right)=\left(q\left(r\right),z\left(r\right)\right):\;1\leq r\leq N_{r}\right\}$$

where $N_{r}$ is the number of projected representative points.

**Figure 2:**
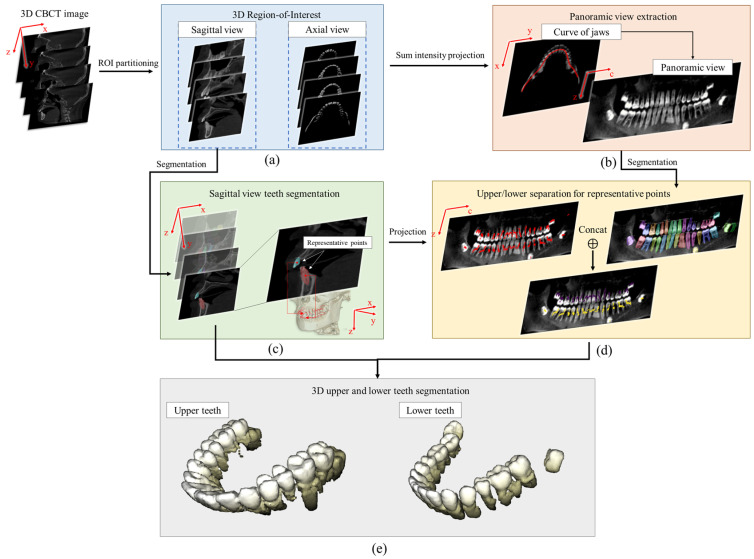
Schematic diagrams of 3D upper and lower teeth segmentation method for CBCT images. (**a**) Two view direction considered from CBCT; (**b**) exacting the panoramic view from the defined jaw curve; (**c**) teeth segmentation along the sagittal view; (**d**) using the teeth segmentation on the panoramic view to separate the upper and lower teeth; (**e**) example of separated upper and lower teeth.

Third, considering cells $px_{pano_{upper}}\in \;{M_{pano}}_{upper}$, if $p\left(r\right)\in px_{pano_{upper}}$, the segmented mask $\;{M_{sagittal}}_{n}\left(s\right)$, which is represented by p(r), is collected in the group of upper teeth masks ${\tilde{X}}_{upper}$. Similarly, if $p\left(r\right)\in px_{pano_{lower}}$, the segmented mask $\;{M_{sagittal}}_{n}\left(s\right)$ is collected in the group of lower teeth ${\tilde{X}}_{lower}$; the obtained results are shown in Figure 2.

### 3.2. Teeth Segmentation from the Scan Images of Plaster Dental Models

Considering scan images of plaster dental models as input data for teeth segmentation, the color of voxels $s=\left(h,s,v\right)\in {\mathbb{N}}^{3}\;:1\leq h\leq N_{h},\;1\leq s\leq N_{s},\;1\leq v\leq N_{v}$, where $N_{h}$, $N_{s}$, and $N_{v}$, are values in the HSV color space, respectively in channels h (hue), s (saturation), and v (brightness), which is considered as the main feature for separating the teeth section from the gum part. The fixed radius nearest neighbor (FRNN) is applied to classify each voxel into two groups with labels 1 and 2 representing the group of teeth and gum, respectively.

The training dataset $S_{all}=\left\{\left(s_{1},\varphi_{i}\right),\left(s_{2},\varphi_{2}\right),\ldots ,\;\left(s_{n},\varphi_{n}\right)\right\}$, where $\varphi_{i}\;\in \;\left\{1,\;2\right\},1\leq i\leq N_{voxel}$, $N_{voxel}$ denotes the number of voxels in the training pattern and is constructed from a dataset of scan images of plaster dental models. Based on this training dataset, with $s_{input}$ as a voxel input to the FRNN model, the steps for evaluating the label for this voxel are as follows:

Step 2-1: The first step is to select candidates around $s_{input}$ as $S_{can}$ through FRNN, as stated in Equation (7).

(7) $$FRNN\left(s_{input},S_{all},R\right)=\left\{s_{can}\in S_{all},d\left(s_{input},s_{can}\right)<R\right\}$$

where R is a constant obtained from the developer.

Step 2-2: Then, the distance between $s_{input}$ and candidates is calculated based on the simplified law of gravitation, as stated in Equation (8).

(8) $$F\left(s_{input}\right)=\underset{i=1,2,\;s_{can_{k}}\in S_{can}\;}{\sum }\varphi_{i}D\left(s_{input},s_{can_{k}}\right)$$

where D( · ) is derived from the formula for gravitation [48].

**Figure 3:**
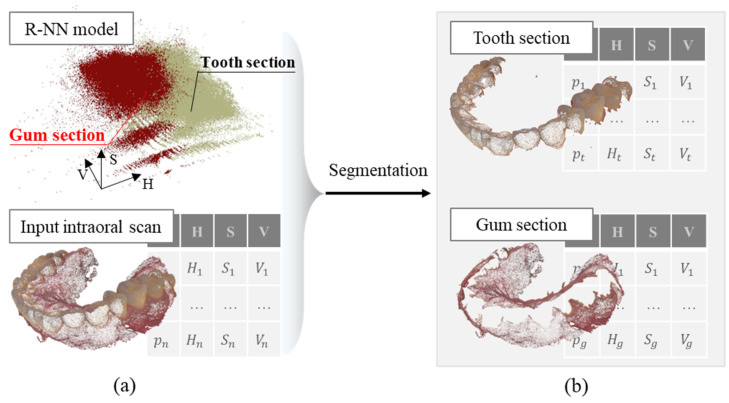
Schematic diagrams of teeth segmentation method using the scan images of plaster dental models. (**a**) The intraoral scan data input to the RNN model for teeth–gum segmentation; (**b**) Example of teeth–gum segmentation results.

Step 2-3: Finally, a decision is made according to the sum of the gravitational forces of all $s_{input}$-candidate pairs. If $F\left(s_{input}\right)<0$, the label $\bar{\varphi }$ of the $s_{input}$ is set to 1. Otherwise, $\bar{\varphi }$ is set to 2. This process is repetitively applied to every voxel in scan data separating input data into two groups of voxels shown in Figure 3.

### 3.3. Teeth Detection and Identification on 2D Maximum Intensity Projection (MIP)

This process aims to obtain the position and identification information for each individual tooth from the axial MIP view. The 3D teeth model, which has various orientations depending on the structure of the patient’s mandible bone, especially in cases of lower teeth, must be aligned along the *y*-axis to obtain the optimal axial viewpoint after projection.

Step 3-1: To rotate the segmented 3D teeth model, the angle α(MeGo, THP), which lies between the line MeGo representing the inclination of the mandible bone and vertical axis, is utilized as the rotation angle. This angle can be measured automatically by adopting the method developed by Ahn et al. [38].

Step 3-2: Maximum intensity projection ${ℳ}_{X_{upper}}$ of the upper jaw and ${ℳ}_{X_{lower}}$ of the lower jaw along are used to detect and identify teeth based on $X_{upper}=X⊙\;{\tilde{X}}_{upper}$ and $X_{lower}=X⊙\;{\tilde{X}}_{lower}$, respectively, which are stated as follows:

(9) $${ℳ}_{X_{upper}}=\underset{z}{\max}X_{upper}\left(x,y,z\right);\;\;\;\;{ℳ}_{X_{lower}}=\underset{z}{\max}X_{lower}\left(x,y,z\right)$$

This procedure is necessary for teeth segmentation on each MIP. Considering ${ℳ}_{X_{upper}}$, the segmented masks ${M_{X_{upper}}}_{n}$ are represented by the center point $p_{\;{M_{X_{upper}}}_{n}}$ of the detected bounding box $\left({x_{X_{upper}}}_{n},{y_{X_{upper}}}_{n},{h_{X_{upper}}}_{n},{w_{X_{upper}}}_{n}\right)$. The coordinates of the representative point $p_{\;{M_{X_{upper}}}_{n}}$ are stated in Equation (10).

(10) $$p_{\;{M_{X_{upper}}}_{n}}\left(x,y\right)=\left({x_{X_{upper}}}_{n}+\frac{{h_{X_{upper}}}_{n}}{2},{y_{X_{upper}}}_{n}+\frac{{w_{X_{upper}}}_{n}}{2}\right)$$

Similarly, the $p_{\;{M_{X_{lower}}}_{n}}$ is obtained as the representative point for the segmented teeth of the lower jaw.

Step 3-3: The midline of the upper jaw is defined as the point ${p_{C_{X_{upper}}}}_{mid}$ on the jaw profile curve $C_{X_{upper}}$ that has the largest distance to the centre point $C_{X_{upper}}$. To calculate this midline, after obtaining $C_{X_{upper}}$, which is defined in Equation (11) and extracted as the outer boundary of the upper jaw MIP, the distance between r(c) and $C_{X_{upper}}$ is calculated.

(11) $$C_{X_{upper}}=\left\{r_{X_{upper}}\left(c\right)=\left(x\left(c\right),y\left(c\right)\right):\;1\leq c\leq N_{c_{upper}}\right\}$$

where $N_{c_{upper}}$ is the number of points on the upper curve.

Then, ${p_{C_{X_{upper}}}}_{mid}$ is defined as stated in Equation (12).

(12) $${p_{C_{X_{upper}}}}_{mid}\left(x,y\right)=\left(\frac{\sum_{c_{min}\leq c\leq c_{max}}x\left(c\right)}{c_{max}-c_{min}},\frac{\sum_{c_{min}\leq c\leq c_{max}}y\left(c\right)}{c_{max}-c_{min}}\right),\;\;d\left(r_{X_{upper}}\left(c\right),\;C_{X_{upper}}\right)\geq \underset{c}{\max}\;d(r_{X_{upper}},C_{X_{upper}})-\Delta d,\;c\in \left[c_{min},\;c_{max}\right]$$

where Δd is the threshold factor for locating the points with the largest distances to the centre $C_{X_{upper}}$. This step is also applied to the lower jaw $X_{lower}$ to define the lower midline, known as the vector between ${p_{C_{X_{upper}}}}_{mid}$ and $C_{X_{upper}}$.

**Figure 4:**
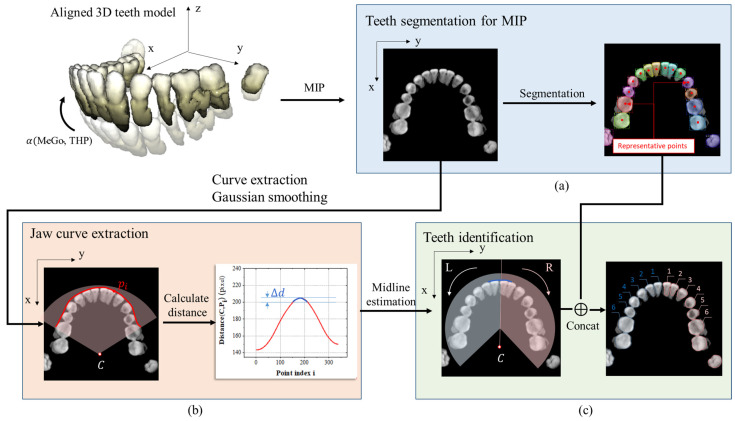
Schematic diagram of teeth detection and identification processes. (**a**) Teeth segmentation results on the MIP view along the axial direction. (**b**) Extracting the jaw curve for detecting the midline. (**c**) Numbering teeth based on the detected midline.

Step 3-4: Concatenating with the obtained midline, segmented teeth can now be split into the left and right teeth groups. According to the Federation Dentaire Internationale (FDI) system, each tooth in each group is assigned a number for identification. For each group, the central and lateral incisors are numbered 1 and 2, respectively. Continuously, the canine and 1st and 2nd premolars are annotated with numbers 3, 4, and 5, respectively. Finally, the 1st molar is identified as the 6th tooth, as shown in Figure 4.

To apply this process to scan images of plaster dental models, in Step 3-2, the depth map converted from the teeth section obtained after segmentation can be used to replace the MIP from the upper and lower teeth.

### 3.4. Key Points Detection for Parameters

The regions-of-interest (ROIs) of the left and right teeth groups are cropped from the MIP of each jaw from the segmentation and identification results. From the obtained ROIs, two landmarks to measure mesio-distal widths in all teeth need to be located. The accuracy of this task should be sufficiently high to obtain the finest tooth size measurement results. Therefore, we used a decentralized CNN-based model previously proposed as a high-accuracy landmark-detection method for medical images. The advantage of this method is that it narrows the ROI according to each order, which not only reduces the number of unrelated features that can affect the results but also improves the diversity of the training dataset.

The weight factors of the trained CNN models are adjusted based on the difference between the output, $A^{i}$, obtained from the deep-learning model containing the calculated position value, i, for the input image, and the label, $Y^{i}$, created based on the actual position of the required points in the input image. The difference in L is calculated using the mean square error loss function stated in Equation (13), which is useful for handling features corrupted by outliers, where *n* denotes the number of labels in the dataset.

(13) $$L=\frac{\sum_{i=1}^{n}{\left(A^{i}-Y^{i}\right)}^{2}}{n}$$

After each training step, the weight factor, $\theta_{t}$, is updated for the ${\left(t+1\right)}^{th}$ iteration based on the rule stated in Equation (14), where m is the batch size (m=64), η is the learning rate (initially set as 0.001 and updated using Stochastic Gradient Descent with Momentum (SGDM) (momentum = 0.95) [49]), and ∇ is the gradient operator.

(14) $$\theta_{t+1}=\theta_{t}-\eta \nabla_{\theta_{t}}\left[L\left(A_{m},Y_{m}\right)\right]$$

**Figure 5:**
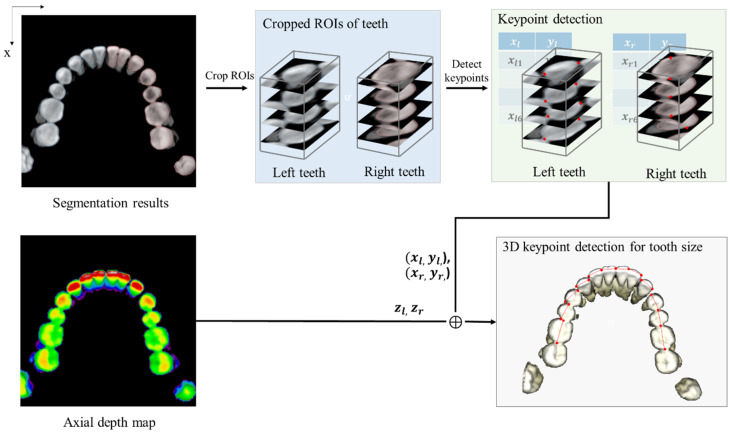
Schematic diagrams of 3D key points detection for measuring tooth size.

**Figure 6:**
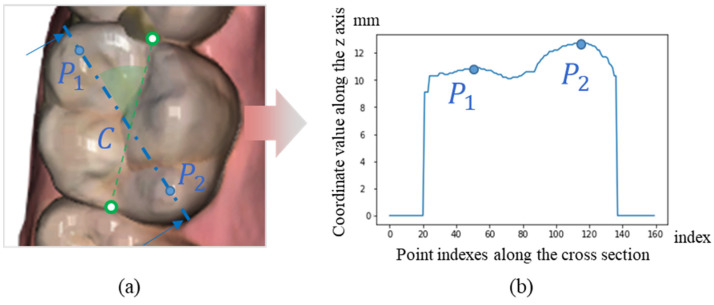
Depth profile extracted from the scan images of plaster dental models with cusp tips. (**a**) The considered cross section for checking the z coordinate values. (**b**) Demonstration of z-value profile along the cross section and the position of $P_{1}$ and $P_{2}$ as peak points.

Subsequently, after receiving the 2D landmark coordinates $(x_{l,}$ $y_{l,})$ $(x_{r,}$ $y_{r,})$ detected on the ROIs cropped from the MIP of each jaw, the coordinate values $z_{l,}$ $z_{r}$ along the axial view are collected by referring to the 2D acquired positions on the axial depth maps projected from $X_{upper}$ and $X_{lower}$ along the axial direction. Finally, the mesio-distal widths of teeth are calculated as the 3D distance between the two corresponding landmarks, as shown in Figure 5. The identification of these landmarks is done by applying a decentralized CNN to the 2D image of each tooth, which is cropped from the segmentation result of the overall image. From there, the 3D coordinates are extracted by comparing the 2D coordinates and the depth value from the axial depth map. Additionally, the 2nd parameter, known as maxillary arch width, requires the cusp tips to be located on specific teeth, namely, canine, 1st premolar, and 1st molar on the scan images of plaster dental models. From the ROI cropped from the depth map of this data, based on the detected key-point for tooth-size parameter, a depth profile along the bucco-lingual direction is extracted from the depth map, in which cusp tips are located as the peak points as shown in Figure 6. 3D positions of the cusp tips are gathered and used to calculate the distance parameters according to each specific tooth.

## 4. Results

### 4.1. System Implementation

**Figure 7:**
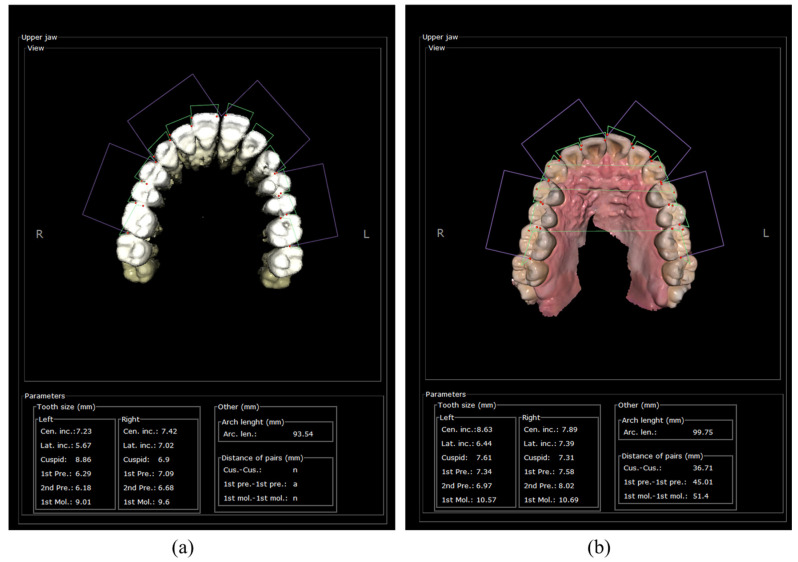
Designed GUI of the developed function for CBCT (**a**) and the scan images of plaster dental models (**b**).

A graphical user interface (GUI) is developed based on the framework of a previous study conducted by Ahn et al. [38] to conveniently utilize this function. After inputting the original CBCT images and running the previous functions to detect the necessary cephalometric landmarks, users can activate the developed function by clicking a button. The measurements of tooth size, including visualization and parameter calculation, can be easily observed in the GUI, as shown in Figure 7. In particular, the green lines connecting the landmarks detected as red dots in the figures of the upper and lower jaw represent tooth size shown in the frame of parameters. The designed GUI allows the user to adjust the landmarks by manually clicking the correct positions of specific landmarks and recalculating the related parameters based on the obtained analysis results. The purpose of this option is to allow users to modify the unsatisfactory detection results. The total operation time for the AI-based analysis is approximately 30 s for CBCT and 5 s for the scan images of plaster dental models in our test configuration that uses a Core i7-11700 with 64 GB of random-access memory and a GeForce RTX 3070 (NVIDIA) graphics processing unit.

The GUI was designed based on orthodontists’ feedback to ensure ease of use and alignment with their workflows. Although formal usability testing has not been conducted, the design was iteratively refined through expert input. Future plans include broader usability testing to further enhance its practicality.

### 4.2. Inter-Observer and Intra-Observer Analysis

The developed program is validated via manual measurements as the gold-standard in practice. In this study, the measurements are conducted by two experienced orthodontists with more than 10-year working experiences in this field. Two human experts measured tooth size on plaster models with digital calipers. An orthodontist measured tooth size twice at two-week intervals to validate the reliability of the intra-observer. After the validation process, the intraclass correlation coefficients (ICCs) and 95% confidence interval (95% CI) for intra-observer reliability of measurements were 0.9924 (0.9912–0.9934) for observer 1 and 0.9953 (0.9945–0.9959) for observer 2. The combined ICCs for intra-observer reliability were 0.994. The inter-observer in this research consisted of three measurements including the automated values generated by the developed method and the means of two manual measurements conducted by two human experts. The ICCs and 95% CI for inter-observer reliability were 0.9238 (0.9131–0.9333) for the CBCT data and 0.968 (0.9501–0.9773) for the scan images of plaster dental models. The mean absolute error (MAE) was less than 1 mm for all measurements shown in Table 1.

To further demonstrate the consistency between the measurements of the proposed method and those obtained by the two human experts, analysis of variance (ANOVA) tests were conducted on the validation data for the following hypotheses (*F_dis_* = 2.68, *α* = 0.05):

1. Null hypothesis $H_{o}$: all measurements are equal $\mu_{1}=\mu_{2}=\mu_{3}=\mu_{4}$.
2. Alternative hypothesis $H_{\alpha }$: At least one measurement is unequal.

The F values of the tooth-size parameters in 30 cases were smaller than the standard $F_{dis\;}$ extracted from the table. All collected *p*-values were larger than 0.05. Therefore, there was no statistically significant evidence against the null hypothesis $H_{o}$, demonstrating the consistency between the measurements of the proposed method and those obtained by the human experts.

### 4.3. Measurements of Tooth Size

**Figure 8:**
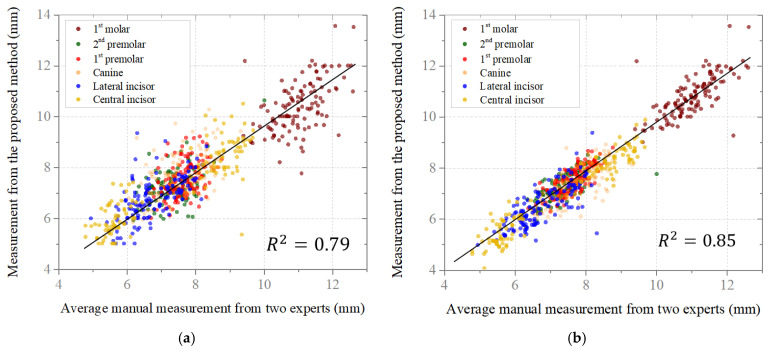
Scatterplots of correlations between manual measurements and those obtained by the proposed method for tooth size: (**a**) CBCT images; (**b**) scan images of plaster dental models.

To clearly illustrate the correlation between the measurement results of the developed program and those obtained by the two human experts, scatterplots of the correlations between tooth size measurements for different tooth types are shown in Figure 8. Two human experts measured every case twice; therefore, the values representing the manual measurements in the plots denote the means of four individual measurements. In CBCT, the correlations between the manual and automated measurements of 1st molar are lower than those of the other cases based on this scatterplot. The accuracy of molar measurements is affected by the dataset collection process during CBCT imaging. If the upper and lower teeth are not properly separated, especially for molars with horizontally spread crowns, the lack of a clear boundary due to the 0.3 mm resolution can hinder segmentation and reduce measurement precision.

**Figure 9:**
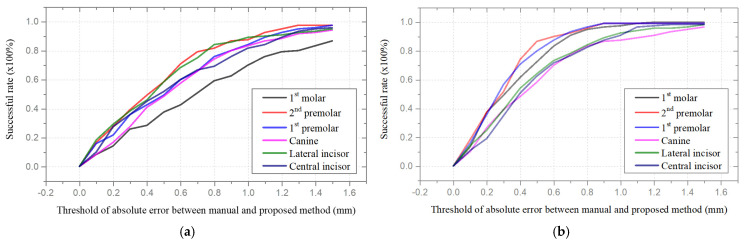
Success rate of tooth size for six types of teeth: (**a**) CBCT images; (**b**) scan images of plaster dental models.

In terms of the absolute errors between manual and the proposed methods, the success rate corresponding to each tooth type is investigated, which represents the percentage of test cases with deviations between manual and automatic measurements smaller than a considered threshold. With 30 test cases with upper and lower jaws for each case, for a range of the considered threshold from 0 to 1.5 mm, the success rates are shown in Figure 9. The tendency of the success rates for different teeth types in the CBCT data is shown in Figure 9a. The results for most teeth reached 0.8 with a threshold smaller than 1 mm. However, the measurement of the 1st molar requires a 1.2 mm threshold to reach a success rate of 0.8, coinciding with the results of the previous analysis. Regarding the scan images of plaster dental models, there is a significant enhancement in term of measurement accuracy. The success rate of all teeth measurements reached 0.8 with a threshold smaller than 0.8 mm shown in Figure 9b. In particular, the measurement of the program for the 1st molar, 1st premolar, and 2nd premolar achieved a success rate of 0.8 with a threshold smaller than 0.6 mm.

### 4.4. Measurements of Arch Widths

The measurements of the developed program were also compared with the manual measurements by two orthodontists in arch widths. The scan data of plaster dental models were only considered as input data for measuring these parameters because of poor tooth crown resolution of CBCT images. The MAE between the proposed method and the means of two measurements by two orthodontists for the canine and 1st premolar was approximately 0.6 mm. These errors in the 1st molar are slightly higher with the MAE of around 1.08 mm as per Table 2.

**Figure 10:**
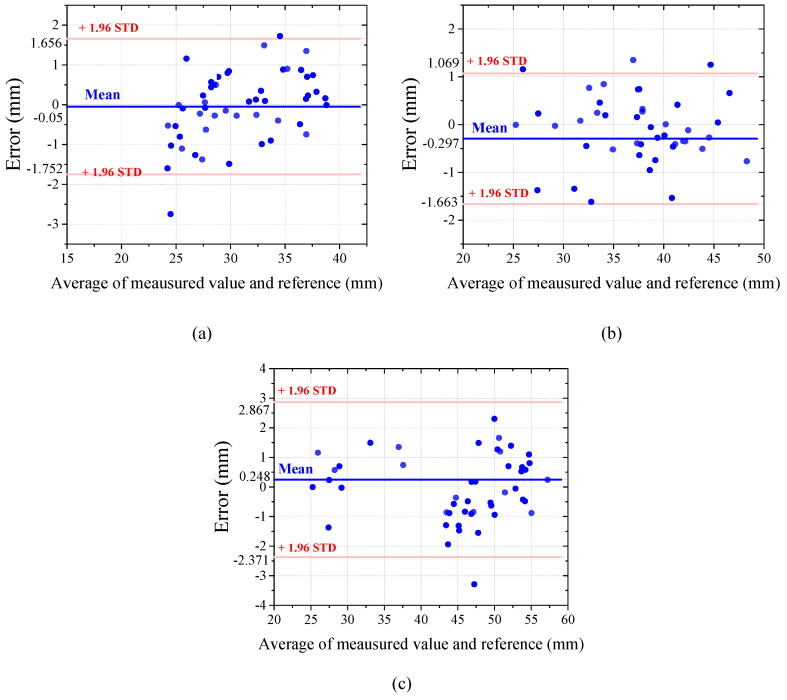
Bland–Altman plots comparing arch widths measured by manual and the proposed method: (**a**) canine; (**b**) 1st premolar; (**c**) 1st molar.

The results of the methods were visualized via Bland–Altman (B–A) plots for all teeth in Figure 10, which includes the horizontal lines of mean difference (red solid line) and mean difference ± 1.96 standard deviation (STD) (blue solid lines). The error range of measurements in the 1st molar is larger than that of the other teeth. This can be caused by the characteristics of the 1st molar, which is significantly different from the canine and 1st premolar, with larger cusp tips. Therefore, it can lead to wider deviation in detecting key-points on the cusp tips.

### 4.5. Performance of Tooth Identification

**Figure 11:**
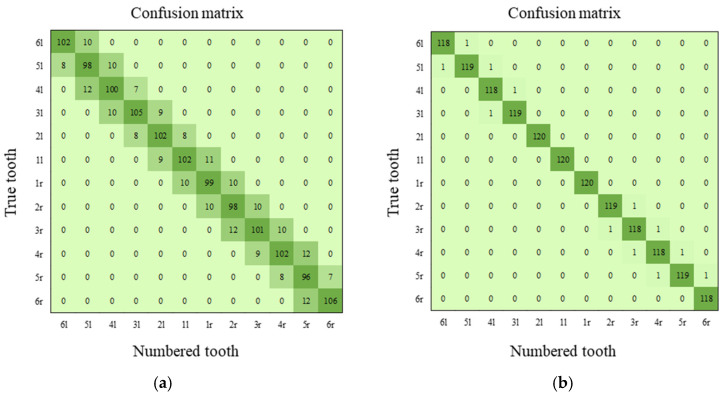
Confusion matrix for tooth identification: (**a**) result of the midline-fixed method; (**b**) result of the proposed method.

A method to automatically estimate the midline of the jaw on the MIP is introduced in Step 3-3. Confusion matrices for the two methods are generated to demonstrate the efficiency of the proposed method compared to using the y value of $C_{X_{upper}}$ and $C_{X_{lower}}$ as the fixed midline in Figure 11. As shown in Figure 11a, the numbers given to the teeth can be shifted by one or two units for both sides because there is an inevitable deviation between the patient’s head orientation and Oxyz coordinate and the clinical variety of rotation of jaws on the Oxy, which causes a chain reaction of wrong identification for all teeth from midline to both sides. The midline can now be adaptively evaluated based on the detected jaw arch curves with the proposed method, which significantly improves the individual tooth identification in Figure 11b.

### 4.6. Effect of Tooth Abnormal Arrangement and Rotation

The method proposed in this study measures tooth size based on Bolton’s ratio. However, Bolton’s ratio assumes that all teeth are arranged in an ideal tooth-by-tooth order, which is not guaranteed in practical cases. There are cases in which teeth are arranged irregularly with the random teeth deviating from the line of occlusion; consequently, they overlap with nearby teeth on the panoramic view. In addition, for other cases, a random tooth can also have an abnormal rotation along the *z*-axis.

**Figure 12:**
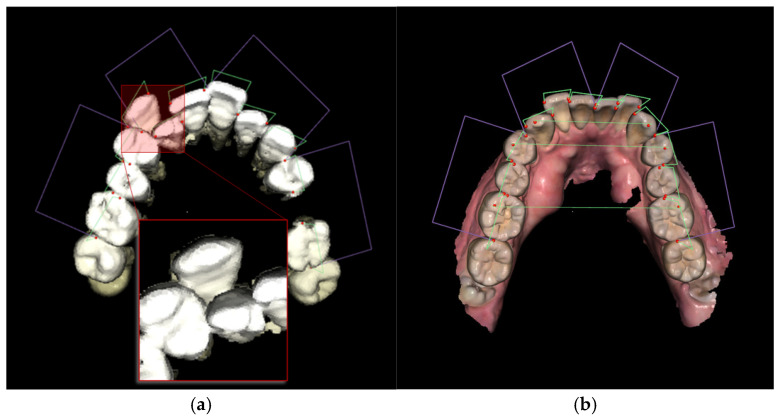
Example for solved abnormal test cases: (**a**) Abnormal teeth arrangement based on CBCT image; (**b**) abnormal teeth arrangement based on the scan image of the plaster dental model.

With an approach that focuses on extracting the upper and lower teeth and sequentially detecting the 3D tooth size instead of extracting each individual tooth, the proposed method can function in these unanticipated situations. In addition, detecting key-points using the decentralized CNN for each tooth overcomes the problem related to the uncommon *z*-axis rotation of random teeth. This advantage is demonstrated in both the CBCT and scan images of plaster dental models shown in Figure 12.

### 4.7. Effect of Metal Artefacts in CBCT Images

**Figure 13:**
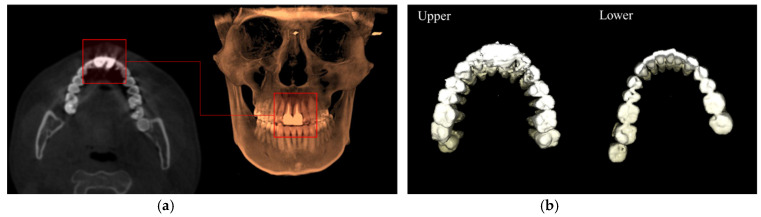
Example for solved CBCT cases with a metal artefact: (**a**) Metal artefact; (**b**) 3D upper/lower segmentation.

Metal artefacts significantly affect the analysis of CBCT through the incorrect Hounsfield value for the amount of voxels in the CBCT data, which causes difficulties when discretizing tissues shown in Figure 13a.

In spite of this effect, the proposed method optimizes the ability to define the boundary between the upper and lower teeth with the 3D segmentation of the upper and lower teeth along the sagittal direction instead of the axial direction as conventional methods. Moreover, no empty space splitting occurs between two sections when patients strongly close their month in Figure 13b.

On the other hand, the accuracy of tooth size measured using the 3D segmentation that results from CBCT images is inevitably affected by metal artefacts. This point can be overcome by utilizing the scan images of plaster dental models, which are immune to the metal artifact effects.

## 5. Discussion

Aiming to measure the mesio-distal tooth width by the proposed method from the central incisor to 1st molar of both the left and right upper and lower arch is, first, a 3D segmentation of the upper and lower teeth along the sagittal axis by combining the segmentation results from the panoramic view. Second, the midline of the jaw is detected based on the arch curve from the MIP, which provides the standard for individual tooth identification. Third, the key points representing the mesio-distal width of the tooth are sequentially detected, which satisfies the requirements for measuring tooth size. The accuracy of the proposed method was compared with the manual measurement results from two orthodontists. The MAE and the correlation coefficient R^2^ between automatic and manual measurements for tooth-size are respectively 0.49–0.78 (mm) and 0.79 in the case of using the CBCT; 0.32–0.53 (mm) and 0.85 in the case of using intraoral scan data. In term of the arch widths measured on intraoral scan data, the MAE between two validation measurements is from 0.55 to 1.08 mm.

Jang et al. [39] introduced an interesting method for 3D individual tooth segmentation using CBCT images, which can be used for measuring tooth size. The first concept is the segmentation of the panoramic view, which is used for tooth identification. Each individual tooth is continuously segmented along the *z*-axis based on the combination of segmentation mask boundaries and jaw arch curve. Accordingly, individual 3D tooth models are obtained. However, the disadvantage of this method is that its identification and segmentation performance are highly dependent on the tooth arrangement. Notably, teeth sometimes do not follow the ideal state in clinical cases requiring treatment. Therefore, overlapping teeth in a panoramic view affect the results technically and significantly. Moreover, overbite and overjet in dentition of CBCT images make their identification and segmentation performance difficult. However, the proposed method is proven to be able to handle these cases as shown in the previous section.

Tian et al. [50] and Xu et al. [51] proposed other interesting methods for the automatic classification and segmentation of teeth based on intraoral scanned data, which can also be used for measuring tooth size. Although it can segment the teeth from 3D input data and classify the type of teeth, the intraoral scanned data are considered to be naturally convenient for this task compared to CBCT images. During the scanning process, orthodontists conduct surface data collection for the upper and lower jaws sequentially. Thus, the 3D data obtained from these jaws are initially separated according to the operation process. In addition, the intraoral scanned data simply contains data related to the surface of scanned teeth and gums, unlike CBCT data, which has the form of a digital box with multiple voxels of various tissues including bone, flesh, skin, et al. Therefore, there is no process for extracting the teeth section from the original CBCT images in previous studies. Obviously, selecting intraoral scanned data as the applied target decreases the complexity of the required work compared to the solution applicable for both the CBCT and intraoral scan data which is proposed in this study.

Yu et al. [52] demonstrate the effectiveness of an AI-driven system in measuring tooth dimensions, offering comparable accuracy to traditional manual digital methods. Using machine learning for tooth segmentation and automated analysis, the AI system achieved high reliability and reproducibility with minimal deviations and significantly reduced measurement time by over 50%. This highlights its potential to streamline orthodontic diagnostics, providing accurate, consistent results while enhancing efficiency in clinical workflows. However, the application of this method is mainly aimed at intraoral scan data.

Smith et al. [53] explores the application of artificial intelligence in dental morphology. The researchers employed a hybrid model combining K-means clustering and graph-based variational autoencoders to analyze tooth dimensions. Measurements were taken using Vernier calipers to determine the mesiodistal and buccolingual widths of extracted teeth. The AI model effectively categorized tooth shapes and sizes into three distinct clusters, facilitating tailored dental interventions. The study underscores the potential of advanced AI techniques in enhancing personalized dental diagnostics. However, analyzing the medical images to measure the tooth size directly is not included in the scope of this research.

Although the performance of the proposed method has been validated, there are several limitations to the current research. First, the number of test cases—limited to 30—is relatively small and may not sufficiently demonstrate the practical applicability of the method. Future studies will aim to expand the dataset and incorporate expert feedback to not only validate the method’s broader applicability but also enhance the user experience. Second, during data collection, specific patient conditions such as overbite and overjet were not recorded. As a result, the impact of these conditions on the developed method has not been investigated, necessitating further studies to address this limitation. Third, the current method does not support the extraction of 3D models for individual teeth, which could potentially improve the accuracy of tooth-size measurements in a 3D environment. Addressing this limitation will be a key focus for future developments in this research.

## 6. Conclusions

This study presents an innovative solution for measuring tooth size and arch widths using CBCT data and scan images of plaster dental models through an intelligent and automated process. The proposed system integrates deep learning models for 3D segmentation, individual tooth identification, and key-point detection, enabling precise and efficient parameter calculations. Key advantages of the system include its ability to automate traditionally labor-intensive tasks, significantly improving productivity by reducing processing time compared to manual methods, and its compatibility with both CBCT and scan images, ensuring flexibility for various clinical setups. Additionally, the system features an interactive graphical interface with advanced 3D visualization, making it easier for users to observe teeth arrangements and extract parameters, offering functionality comparable to commercially available software.

## Figures and Tables

**Table 1:** Deviation between the measurements of the proposed method and those of manual measurements conducted by two orthodontists.

No.	Tooth Name	Mean ± 1.96 × STD	MAE for CBCT Images		MAE for the Scan Images of Plaster Dental Modes	
1	Central incisor	7.25 ± 3.30	1st rater:	0.61	1st rater:	0.46
			2nd rater:	0.58	2nd rater:	0.47
2	Lateral incisor	6.74 ± 1.45	1st rater:	0.52	1st rater:	0.46
			2nd rater:	0.51	2nd rater:	0.46
3	Canine	7.63 ± 1.14	1st rater:	0.61	1st rater:	0.53
			2nd rater:	0.62	2nd rater:	0.48
4	1st pre-molar	7.66 ± 0.91	1st rater:	0.55	1st rater:	0.33
			2nd rater:	0.55	2nd rater:	0.32
5	2nd pre-molar	7.36 ± 1.10	1st rater:	0.49	1st rater:	0.32
			2nd rater:	0.5	2nd rater:	0.33
6	1st molar	11.03 ± 1.40	1st rater:	0.72	1st rater:	0.36
			2nd rater:	0.78	2nd rater:	0.38

**Table 2:** Deviation between the proposed method and manual measurements conducted by orthodontists in terms of arch widths.

No.	Tooth Name	Mean ± 1.96 × STD	Comparison Results		
			Rater	MAE	STD of AE
1	Canine	31.03 ± 9.44	1st rater:	0.70	0.57
			2nd rater:	0.65	0.54
2	1st premolar	38.88 ± 9.11	1st rater:	0.67	0.46
			2nd rater:	0.55	0.43
3	1st molar	50.40 ± 9.55	1st rater:	1.08	0.81
			2nd rater:	1.08	0.84

## Data Availability

No new data were created or analyzed in this study.
